# Effectiveness of a diabetes prevention program for rural women with prior gestational diabetes mellitus: study protocol of a multi-site randomized clinical trial

**DOI:** 10.1186/s12889-018-5725-x

**Published:** 2018-06-28

**Authors:** Jia Guo, Yujia Tang, James Wiley, Robin Whittemore, Jyu-Lin Chen

**Affiliations:** 1 Xiangya School of Nursing, Central South University, Changsha, Hunan People’s Republic of China; 2 Philip R. Lee Institute for Health Policy Studies and Department of Family and Community Medicine, University of California, San Francisco, CA USA; 3 School of Nursing, Yale University, West Haven, CT USA; 4 School of Nursing, University of California, San Francisco, CA USA

**Keywords:** Woman, Gestational diabetes, Rural area, Prevention, Type 2 Diabetes Mellitus, Cognitive behavioral therapy, RCT

## Abstract

**Background:**

In China, about 53.4 million women (11%) have type 2 diabetes (T2DM). Women with prior 2 (GDM) are at a high risk for T2DM. Postpartum lifestyle interventions have been effective in reducing T2DM for this population, but the evidence is limited to interventions provided in urban areas, despite the fact that a higher prevalence of undiagnosed T2DM was found in rural areas in China. The primary purpose of this proposed study is to examine the effect of a postpartum intensive lifestyle modification (ILSM) program on physiological health outcomes (T2DM development, insulin resistance, and weight-related variables), weight-related health behaviors (dietary intake and physical activity), and psychosocial outcomes (self-efficacy, perceived stress, social support, and health-related quality of life) compared to usual care at 3, 6, and 18 months post baseline assessment. The secondary outcomes are to identify potential mediators and moderators on change of physiological health outcomes.

**Methods/design:**

A multi-site randomized clinical trial (RCT) will be conducted to examine the efficacy of an evidence-based Intensive Lifestyle Modification (ILSM) program compared with usual care for women with prior GDM living in rural areas in China. A total sample of 256 participants will be recruited in the study. The intervention consists of six bi-weekly in-person group sessions, five bi-weekly telephone consultation sessions, and three monthly telephone consultations to encourage behavior change. The usual care provided to the control group will utilize current clinical guideline and recommendations for T2DM prevention. Outcome measures include physiological variables (OGTT-2 h, HbA1c, weight, and waist circumference); weight-related health behavioral (dietary intake and physical activities); and psychosocial variables (self-efficacy and social support) at 3-, 6- and 18- month after baseline. We will also assess the potential cost-effectiveness of ILSM (net cost per T2DM case and per DALY averted) compared with usual care.

**Discussion:**

If successful, this ILSM program can be adapted and used in rural areas as a blueprint for clinical guidelines to decrease T2DM by improving postpartum GDM care in China. Findings of this study are expected to make a significant contribution to public health practice and health policy related to T2DM prevention in China.

**Trial registration:**

Chinese Clinical Trial Registry, ChiCTR1800015023. Registered 1 March 2018 - Retrospectively registered, http://apps.who.int/trialsearch/default.aspx.

## Background

Type 2 Diabetes Mellitus (T2DM) is a global health concern especially in low- and middle-income countries, which constitute 77% of all cases of diabetes. China is also experiencing this global health epidemic because China has the largest diabetic population in the world (about 114 million adults or one in 4 adults) [1, 2] and the overall disease burden of diabetes is huge (19.12 DALYs per 1000 population in 2010) [3]. However, health disparities are seen in rural areas as undiagnosed T2DM prevalence is higher in rural than urban areas in China (70.5% vs. 58.0%), possibly due to lack of awareness of T2DM and access to high quality health programs in rural areas [4]. Thus, efforts to prevent T2DM and effective intervention to prevent T2DM in rural areas are essential in China.

Risk factors associated with T2DM include women with Gestational diabetes mellitus who lives in rural area [5]. Gestational diabetes mellitus (GDM) is defined as any degree of glucose intolerance with onset during pregnancy [6]. The prevalence of GDM is 18.9% in China [7]. For mothers with prior GDM, the overall risk of developing T2DM is over seven-fold higher than those who had normal glyceaemic pregnancies [8]. The incidence of T2DM in 1–10 years after a diagnosis of GDM ranges from 10 to 40% [9], with up to 70% of women developing T2DM within 20 years, depending on race/ethnicity [10]. Because of the recent family planning policy change in China, many women will have more than one child during child bearing age and repeated exposure of GDM (diagnosis of GDM in more than one pregnancy) will significantly increase women’s risk for T2DM [7]. In addition, residents in rural areas are largely unaware of their T2DM risk [4] and health care program for women with prior GDM is absent despite the high rate of undiagnosed T2DM in rural areas [11], an intervention program tailored to this high risk group will be the first step for changing clinical practice and policy development aimed to decrease health inequity in China.

Many interventions, including lifestyle and pharmacotherapy interventions, have been developed to prevent T2DM. Lifestyle interventions, in particular, have demonstrated potential efficacy in reducing T2DM incidence and may be superior to pharmacological retreatment [12]. A recent systematic review [11] examined the efficacy of lifestyle interventions in decreasing T2DM for women with GDM using randomized clinical trials found that the majority of the trials (92%) targeted women in an urban setting [13–23] and the results suggest that postpartum lifestyle interventions for women with GDM history can decrease T2DM risk significantly on the short-term follow up. However, these studies are limited to pilot or feasibility studies and only three studies were conducted in China and all of them focused on women in urban cities [15, 16, 21].

Although about half of the population (700 million people) resides in rural areas in China [24], there is a huge rural-urban gap of income, education and health resources, with the living situation in urban areas being much better than that in the rural areas [25]. People in rural areas are also suffering from unhealthy lifestyle practices as they are more likely to smoke, exercise less frequently, and have lower health literacy than their urban counterparts [26, 27].

Women living in rural area in China practice more traditional Chinese customs of a “sitting month” during postpartum period that include more sedentary behavior and increased consumption of high-calories, high-fat, and high-protein food intake during the first month [28]. These traditional practices might make Chinese women miss the optimal period for weight loss and healthy lifestyle change [29]. Other issues could increase risk for T2DM in women in rural areas include inadequate in-service training, staff shortages, poor resource distribution, and transportation difficulties which create health inequality in rural areas in China [30]. Thus, diabetes prevention interventions with lifestyle modification for rural Chinese women with prior GDM are urgently needed.

The primary purpose of this proposed study is to examine the effect of a postpartum intensive lifestyle modification (ILSM) program on physiological health outcomes (T2DM development, insulin resistance, and weight-related variables), weight-related health behaviors (dietary intake and physical activity), and psychosocial outcomes (self-efficacy, perceived stress, social support, and health-related quality of life)compared to usual care at 3, 6, and 18 months post baseline assessment. The secondary outcomes are to identify potential mediators and moderators on change of physiological health outcomes. This paper describes the development of this culturally sensitive ILSM program and study procedure.

## Methods

A multi-site randomized control clinical study design (RCT) will be used to investigate the efficacy of ILSM on physiological, behavioral and psychosocial outcomes among women with GDM history residing in rural area in China. The study will be conducted in Hunan Province, which is representative of Central South China in geography (mixed plains and mountainous areas), climate (humid subtropical), culture, demographics (Han and multi-minorities), economy, health policy, and lifestyle (diet and physical activities). Two different counties from western and eastern part of Hunan Province (Yongding county in Zhangjiajie and Youxian county in Zhuzhou city) will be recruited as the research sites in order to represent different SES, lifestyle and ethnic groups in Hunan Province. Study participants will be recruited from county-level general hospitals and maternal and children’s hospitals in these two counties. Half of the towns from each county will be selected and assigned at random to the ILSM condition and the rest of the towns to the control condition respectively. Randomization will be done at town level and randomly assign by the study biostatistician using a randomization protocol available on the internet Blinding. Given the nature of the intervention, it is impossible to mask the participants or professionals to the study group assignments. However, data assessors will be blinded to participant study arm and data analysis will be blinded to any potential identifiers (http://stattrek.com/statistics/random-number-generator.aspx).

### Study participants

Participants will be invited to participate in this study if they meet the following inclusion criteria: 1) Women with a history of GDM; 18 years or older; 2) at least six weeks post-partum at the beginning of the study; 3) intend to live in the research counties for at least 3 years; 3) intend to seek primary maternal and child health care in the research sites for at least 3 years; 4) have telephone-access either from family members, friends, or neighbors; 5) be able to speak Chinese.

Exclusion criteria: 1) Planning to become pregnant within the next three years; 2) Currently pregnant; 3) Diabetes diagnosed before pregnancy or after delivery; 4) Medications that influence glucose metabolism; 5) Multiple pregnancies (three or more); 6) Physical or cognitive disability; 7) Current addictive drug abuse; 8) Severe psychiatric disorders.

The power calculations for multivariate procedures are generally more complex and require a priori assumptions that are difficult to verify. Using GPower3.1 we calculated the power of tests of odds ratios in logistic regressions comparing threshold values of OGTT 2 h and HbA1c in ILSM versus control samples. For odds ratios of 0.667 (odds of insulin resistance above threshold in the ILSM groups versus the equal odds in the control group), a combined sample of *N* = 256, relatively high correlations between the other covariates, and a probability of a Type 1 error of .05, the power of the test against null hypothesis of that the odds ratio is 1.0 is about 0.71.

### Recruitment

Recruitment of potential participants will occur by a review of medical records of the women who delivered babies in the research sites. Potential participants who meet inclusion criteria will receive a phone call from a trained project field officer to explain the purpose of the study and assess interest in participation. The trained project field officers who are physicians or nurses working in the research sites will be recruited from the research sites.

The project field officers will confirm their eligibility and obtain consent. As part of eligibility assessment, all interested study participants will undergo postpartum HbA1c testing to make sure the potential subjects do not have T2DM. If HbA1c suggests T2DM, the participant will not be eligible to participate in the study. However, she will be referred to the physicians for further T2DM diagnosis and treatment. If HbA1c are within normal limits, the women will be recruited. Women who do not meet the inclusion criteria will be thanked for their time. We will also document reasons from mothers who decline to participate in the study.

### Intervention

Participants will be enrolled in the study for a total of 18 months. For both groups, the assessment time points include at the beginning of the study (T-0), at 3, 6, and 18 months after baseline assessment (T-1, T-2, and T-3). The 3-, 6- and 18-month after baseline assessment include questionnaires completion as T-0 and the physical assessment (e.g. BMI, waist circumference, blood pressure, OGTT-2 h, and HbA1c). If the OGTT-2 h is abnormal at any time point, the next time point the participants will not assess OGTT-2 h. The timeline of the intervention activities for ILSM group and control group is showed in Table 1.

**Table 1:**
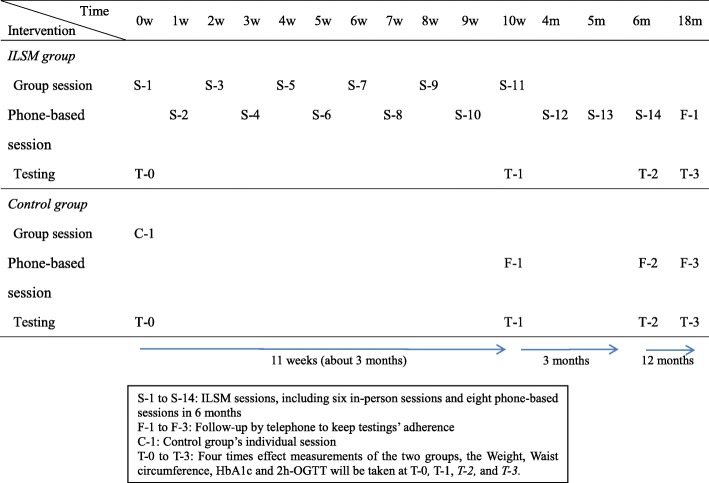
Intervention activities timeline

**Fig. 1:**
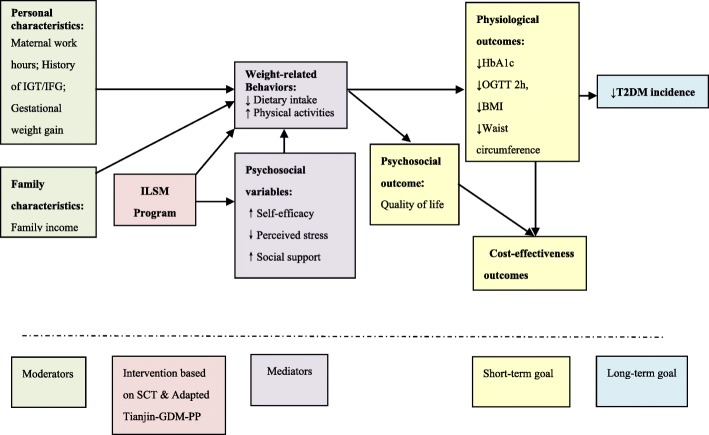
Social Cognitive Theory for Intensive Lifestyle Modifications on Women with prior GDM

ILSM (Intervention):

The ILSM program is guided by the Social Cognitive Theory (SCT) which has been widely applied to the health-promotion and preventive behaviors, and risky lifestyles [31, 32]. The ILSM program is designed to improve behavioral (dietary intake & physical activities), psychosocial (self-efficacy, perceived stress, & social support), physiological outcomes [Hemoglobin A1c (HbA1c), OGTT 2 h, body mass index (BMI), waist circumference, &blood pressure], and psychosocial outcome [quality of life]. Improvement in outcomes will decrease risk of T2DM and health care costs. Figure 1 displays the Social Cognitive Theory model and the proposed intervention.

The ILSM program includes three major components: 1) six bi-weekly in-person group sessions, 2) five bi-weekly telephone consultation sessions, and 3) 3 monthly telephone consultations to encourage behavior change. The ILSM participants who attend bi-weekly in-person sessions at the research sites provided by project field officers will also receive the bi-weekly telephone consultations from them. The time commitment is about 90 min for each in-person session and 20 min for each telephone consultation. The six in-person sessions are group sessions with 10–20 participants. Each participant will receive a brochure of diabetes prevention education information developed by the research team, including diabetes risk factors, healthy eating, exercise, and stress management.

#### Six bi-weekly in-person group sessions

The in-person sessions of ILSM program include six topics on 1) orientation and goal setting, 2) healthy eating patterns, 3) physical activities, 4) stress management, 5) family support on ILSM & family lifestyle patterns, and 6) farewell and relapse prevention. The content of ILSM program sessions are shown in Table 2. Child-care, air-conditioning room, necessary transportation, healthful snacks, and drinks will be provided in each in-person session.

**Table 2:** The topics and content of the six group sessions of the ILSM group

Topics	Content
1st: Orientation and Goals-setting (90 min)	a. Introduction to ILSM. b. Assess personal risk of developing T2DM. c. Inform the life-threatening outcomes of having T2DM. d. Give good examples of T2DM prevention by lifestyle modification. f. Tell and ask the benefits of and barriers to lifestyle change. g. Set goals to achieve and maintain a healthy lifestyle pattern after 3 months. h. Group exercise (30 min).
2nd: Healthy eating patterns (90 min)	a. Assess current eating patterns. b. Summarize the barriers of changing unhealthy eating patterns from the previous two sessions. c. Low Glycemic Index diet. d. Analyze the barrier on dietary and solutions from the previous sessions. e. Help make individual action plan and coping plan on healthier eating patterns. Action plan: What unhealthy eating patterns would you like to change? Pattern 1, 2. For Pattern 1, When? How? Coping plan: Which barrier might prevent you from changing the eating pattern? Barrier 1, 2. How could you overcome this barrier? For Barrier 1, Strategy 1. f. Group exercise (30 min).
3rd: Physical activities (90 min)	a. Assess current physical activities. b. Summarize the barriers of performing physical activities. c. Analyze the barrier’s solutions from the previous sessions. d. Encourage raising more barriers and solutions. e. Help make individual action plan and coping plan on physical activities. Action plan: Which kind of physical activity would you like to perform? Activity 1, 2. For activity 1, When? Where? How long? Coping plan: Which barrier might prevent you from being active at least 2 × 20 min per week? Barrier 1, 2. How could you overcome this barrier? For Barrier 1, Strategy 1. f. Ask to perform aerobics together guiding by a popular DVD disk. g. Group exercise (30 min).
4th: Stress management (90 min)	a. Assess the current stress. b. Stress management: Impact of stress on feelings and behaviors and health; Identify sources of stress; Identify unhelpful stress-management strategies; Discuss more helpful ways of managing stress c. Help make individual action plan and coping plan on mental health management. Action plan: Which kind of strategy would you like to perform to reduce stress? Activity 1, 2. For activity 1, When? Where? How long? Coping plan: Which barrier might prevent you from managing stress? Barrier 1, 2. How could you overcome this barrier? For Barrier 1, Strategy 1. d. Ask to practice some psychological therapy (such as relax exercise) in groups. e. Group exercise (30 min).
5th: Family support on ILSM & family lifestyle patterns (90 min)	a. Assess the current family support on ILSM & family lifestyle patterns (meal-planning and exercise pattern) of each participant family. b. Family healthy eating: Healthier family meal-planning; Dealing with barriers to making healthier choices; Communication strategies c. Family exercise patterns: Importance of family exercise patterns; Suggestions and good examples of family exercise patterns d. Help make family action plan and coping plan with family members, on family support and family lifestyle pattern improvement. e. Ask each family member to express their intentional support to the participant. f. Group exercise (30 min).
6th: Fare well and Relapse prevention (90 min)	a. Assess the current lifestyle and relapse events. b. Relapse prevention: The principle trend phases of behavior changing, Problem-solving strategies for maintaining a healthier lifestyle; Weight and energy balance; Managing relapses c. Emphasize the importance of T2DM screening every six months d. Fare-well and conclusion for the group meeting sessions. e. Group exercise (30 min).

#### Five bi-weekly telephone consultation sessions

To reduce time in traveling and to increase motivation and adherence to behavioral change recommendations, the telephone consultation will occur one week after each in-person meeting. Five telephone consultation sessions of ILSM program include: 1) review of progress toward dietary, physical activity and goals, 2) identify challenges faced in achieving goals, 3) assist with setting new action plans and achievable goals, and 4) encourage achievement of goals.

#### Three monthly telephone consultations at the maintenance phase

After the intensive 11 sessions, the participants will go to the maintenance phase of intervention, which involves telephone consultation sessions on a monthly basis for three months. The maintenance telephone consultation will last no more than 20 min per consultation.

#### Train the trainer

In order to build the sustainability and increase intervention fidelity of the ILSM program, this research uses train the trainer model. The research team will go to each site to train four project field officers in ILSM intervention with lifestyle intervention skills (e.g. ILSM protocols, intervention research, and communication) to provide the ILSM program (Train the trainer model of care) based on the facilitating video and the guidebooks developed by the research team. Their responsibilities will include: (1) Help to identify the eligible participants and recruit the participants; (2) Provide ILSM for the participants; (3) Make the telephone reminders and the site arrangement for the four time-points measurements. Standardization of intervention delivery is assessed by use of the facilitator’s guidebook. Motivational interviewing principles are used with all of the sessions.

#### Usual care (Control group)

Because women with prior GDM are at high risk for T2DM, it is not ethical to provide no treatment. Currently, the clinical guidelines for T2DM prevention include providing information for T2DM prevention to high risk populations, including lifestyle modification and diabetes screening (38). The usual care provided in this proposed project will utilize current clinical guideline and recommendations for T2DM prevention. Each participant will receive the same brochure of diabetes prevention education information as the ILSM group. However, participants in the control group will not have group sessions and phone consultations.

Similar to the ILSM group, four local health workers in the usual care group will be trained as the project field officers. Their responsibilities will include: (1) identify the eligible participants and recruit the participants; (2) ensure participants in the usual care group will receive general oral and written information about awareness of diabetes, information about the importance of dietary modification and physical activity, and suggestions of diabetes screening every three years at the baseline assessment; (3) make the telephone reminders and the site arrangement for the four time-points assessments. The participants will complete the same measurement at the same time intervals at the ILSM groups.

**Fig. 2:**
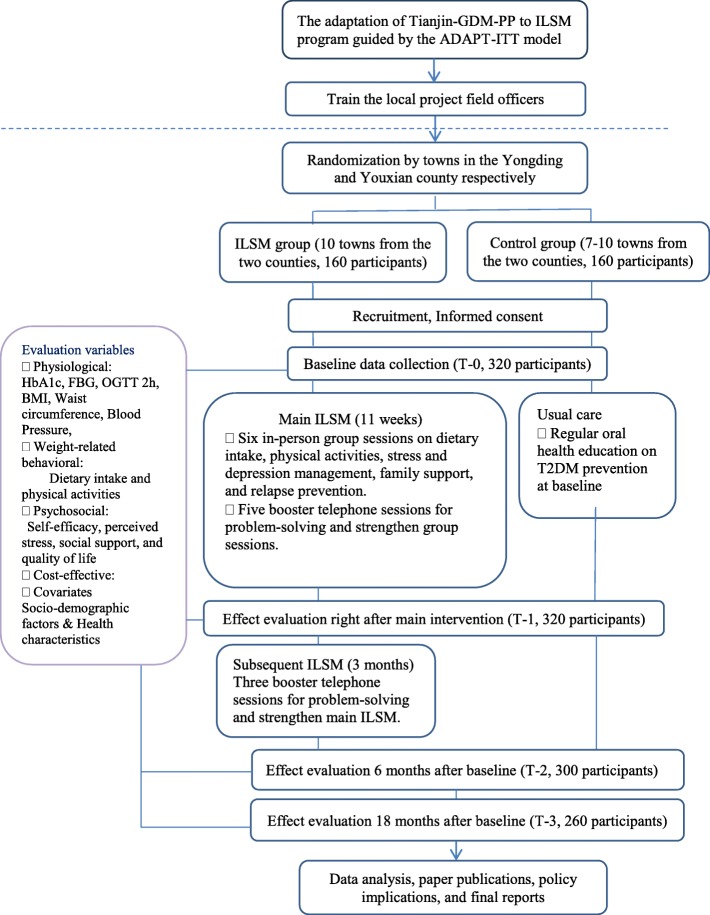
Flowchart of the study

Upon completion of the 18-month study, participants from either the control group or the ILSM group will receive a 100 RMB gift certificate as “thank you” for participation and to sustain their involvement in the study. We will send all participants in this study a birthday message for her birthday. The study flowchart was displayed in Fig. 2.

### Variables and measurements

Each measure was selected to operationalize theoretical constructs and has been used in Chinese rural populations and demonstrated adequate psychometric properties. The research team will measure outcomes at baseline (T-0), immediately after main ILSM at 3 months (T-1), immediately post intervention at 6 months (T-2) and 18-month post intervention (T-3), which are displayed in Table 3. All measures employed in the proposed study will be collected by trained research assistants who are blinded to the group assignments.

**Table 3:** Variables and measures collected at evaluation time points

Variables		Data source	Measures	Time point			
				T-0	T-1	T-2	T-3
Covariates	Socio-demographic factors	Survey & medical record	Age, marital status, education level, occupation, annual household income, and ethnicity.	×			
	Health characteristics		Smoking status, alcohol consumption, pre-pregnancy BMI, gestational weight gain, parental history of diabetes, GDM, and cardiovascular disease.	×			
	Clinical characteristics		Gestational week and age at the diagnosis of GDM, numbers of pregnancies, history of pregnancy complications, birth weight of children, gestation week at delivery, treatment for GDM.	×			
Weight-related Behaviors	Dietary intake	Survey	Food frequency questionnaire [40]	×	×	×	×
	Physical activities		International Physical Activity Questionnaire (Short version) [41]	×	×	×	×
Psychosocial Variables	Self-efficacy: the belief in one’s effectiveness in performing specific tasks.	Survey	Self-rated abilities for health practices scale [42]	×	×	×	×
	Perceived stress: the feelings or thoughts that an individual has about how much stress they are under over a given time period.		Perceived Stress Scale [43]	×	×	×	×
	Social support: the perception and actuality that one is cared for.		Social Support Rating Scale [44]	×	×	×	×
	Quality of life: the general well-being of individuals		The World Health Organization Quality of Life Scale-BREF [45]	×	×	×	×
Physiological outcomes	Insulin resistance: a physiological condition in which cells fail to respond to the normal actions of the hormone insulin	Spot measuring	OGTT-2 h	×	×	×	×
	T2DM screening & development		HbA1c ≥6.5% is considered T2DM.				
Weight-related measures	BMI & Waist circumference	Spot measuring	BMI is weight/height^2^ (Kg/m^2^)	×	×	×	×
Cardiovascular disease risk	Blood Pressure	Spot measuring	Systolic blood pressure, Diastolic blood pressure	×	×	×	×
Cost-effectiveness Outcomes	The costs falling on health and social care systems	Survey & Medical records	A PI-designed questionnaire	×	×	×	×
	The costs falling on individuals and families						
	The effectiveness outcomes		The above clinical and physiological measurements	×	×	×	×

Personal and family characteristics (Covariates): Socio-demographic factors, health characteristics, and clinical characteristics will be collected. Weight-related behaviors (Secondary outcome measures): Two separate questionnaires on changes in dietary intake and physical activity habits will be assessed. Psychosocial variables (Secondary outcome measures): self-efficacy, perceived stress, social support, and quality of life will be measured, the operation definition of which are displayed in Table 3. Physiological outcomes: BMI and waist circumference will be measured as weight-related variables, while blood pressure will be evaluated as cardiovascular disease risk. The biomarkers of insulin resistance will include glucose values on the oral 75 g glucose 2 h post-load glucose tolerance test (OGTT-2 h). T2DM screening and development will be measured by HbA1c. BMI, OGTT-2 h, and HbA1c are the primary outcome measures.

Cost-effectiveness outcomes: We will measure the costs accruing to the health and social care system and to individuals and families for the ILSM program and for health care (in order to assess the effects of ILSM), including inpatient and ambulatory care, medicines, personnel costs to deliver the ILSM, and other procedures and treatments. For the effectiveness component, we will rely on clinical and physiological measurements noted above. These two elements permit cost-effectiveness calculations. In addition, we will assess the costs falling on individuals and families for non-medical costs (travel, child-minding, and time/lost income), which will be used in a secondary cost-effectiveness calculation.

### Statistical analysis

Our analysis plan focuses on comparisons between ILSM and control groups with respect to key intermediate outcomes: HbA1c (%),OGTT 2 h (mmol/L), BMI, waist circumference (in cm), SBP and DBP (mmHg), and measures of dietary intake, physical activity, self-efficacy, and social support. The comparisons take the form of 1) simple differences of average values of change (for quantitative characteristics) from baseline to follow-up measures or turnover tables showing baseline versus follow-up (for data organized into categories) and 2) more complex analyses based on multivariate methods that incorporate adjustment for clustering and covariates.

We will assess cost-effectiveness using an existing spreadsheet-based simulation model called GeDiForCE (Gestational Diabetes Formulas for Cost-Effectiveness), developed by UCSF and Health Strategies International with funding from NovoNordisk. This model uses a decision tree and input values from published studies to portray the risks of T2DM with and without a post-partum intervention. In this study, we will adjust input values to reflect data collected on costs and effectiveness. The cost data described above can be directly incorporated into the model. The effectiveness data will be calculated from the clinical and physiological outcomes above. We will directly incorporate incidence of T2DM. However, due to the relatively short follow-up time frame, as compared with a delay of ten or more years for the incidence of T2DM, we will also use short-term changes in physiological indicators (such as OGTT-2 h and HbA1c) in a simple risk-of-progression model in order to predict long-term effects on incidence of T2DM. This progression model will be calibrated to empirical evidence on the progression from glucose intolerance to T2DM.

## Discussion

Health disparities in T2DM are seen in rural areas in China as undiagnosed T2DM is higher in rural areas than urban [33]. As the new family planning policy launched in China, women with prior GDM will increase their risk for T2DM with subsequent pregnancy. Clinically feasible and sustainable programs aimed to improve healthy lifestyle and decrease T2DM for this high risk group are extremely important for the health of women and their family, especially in rural areas. To our knowledge, this proposed study will be the first to examine the efficacy of a culturally-tailored in-person postpartum lifestyle intervention for women with prior GDM in rural areas in China.

Although current scientific T2DM prevention intervention studies for women with prior GDM have demonstrated some efficacy but data can only be generalized to urban populations [11]. Women with GDM history are at high risk for T2DM and there is a critical policy gap in postpartum care for women with prior gestational diabetes in China. The ILSM with integrative, multidisciplinary approach incorporating clinical, psychological and economic evaluation is expected to make a significant contribution to the development of evidence-based health policy, particularly in postpartum care and diabetes prevention care in rural China.

One study done in Australia reported limited and some success of the phone-based motivational interviewing program in the postpartum period for rural women with prior GDM in Australia [30]. The limited success of the phone-based intervention included that it was difficult to recruit a large proportion of potentially eligible women who may presume their lack of time to exercise with a new baby to care, no time to spend on their own needs or needing return to work early. Based on the literature, it is recommended that intervention builds on stronger theoretical foundation, such as Social Cognitive Theory [17, 20] and Behavioral Theory [23]. The ILSM program based on Social Cognitive Theory (SCT) [34] aims to increase participants’ self-efficacy by setting realistic and achievable goals, providing necessary skills, making individual action plans, improving self-regulation in maintaining and balancing health outcomes and health behaviors, via interactive group sessions and phone consultation provided by a local project field officer. The SCT was demonstrated success in increasing and even maintaining physical activity among individuals with T2DM in several intervention studies [17, 20, 35]. Our intervention is based on the evidence-based Tianjin Gestational Diabetes Mellitus Prevention Program (Tianjin-GDM-PP) [36] in China and results of our preliminary data [37] with rural culture and health care system tailoring.

There are some limitations to this proposed project, including its limited generalizability, self-report data, and limited geographical region only in Hunan Province. Long-term effect of ILSM program to decrease the incidence of T2DM in women with prior GDM will be difficult to show in this proposed project because of the limited project time. To reduce these limitations, we have included the sample size that is theoretically sufficient to detect meaningful clinical difference, used randomization techniques to maximize representation of the target population and increased follow up to 12 month post intervention given the grant funding duration and budget. We also have included several physiological outcomes to minimize self-report bias.

There were a few challenges we have encountered since beginning of this study. These challenges include working with rural health professionals who are less skilled and educated compared with the health professionals in urban cities in China. In the ILSM, the trained project field officers are supposed to be skilled professionals to assure future sustainability and scale-up of this study. The training material and video-facilitating technology were developed to ensure intervention fidelity and easy-understanding and delivery of the ILSM program conducted by the local project field officers, which will also make the program be potential to significantly improve the delivery of care in an increasingly complex healthcare system [38]. A social media chatting group was set up for each arm of each research site separately to bring up questions or concerns. The principal investigator and research assistants are available to answer questions or provide advice regarding with the implementation. Research meetings via telephone are held every month.

Second, since the local project field officers were not trained with research skills, the research team developed several steps to ensure the study is delivered and conducted as it was original planned. Moreover, issues related to recruitment have been identified. Because GDM screening were not widely conducted and recorded in the research sites until the year 2015, only women with a very recent history of GDM can be targeted. Due to high migrant rate to the big cities, only 50% of potential eligible women were still living in the rural areas. In addition, the pressures of caring for a new baby tend to dominate the early postpartum period, the fear of receiving a diagnosis of T2DM, and low risk perception of T2DM are key reasons of refusing to attend the program in this study, which are consistent to the previous literature [30, 39]. Despite the challenges if the intervention is found to be effective, this study would provide important data on the potential efficacy of the intervention and serve as a basis for disseminating the ILSM program to all rural primary care settings and reducing the diabetes related disease burden in mainland China.

## Abbreviations

GDM: Gestational Diabetes Mellitus
GeDiForCE: Gestational Diabetes Formulas for Cost-Effectiveness
ILSM: Intensive Lifestyle Modification
RCT: Randomized clinical trial
SCT: Social Cognitive Theory
T2DM: Type 2 Diabetes Mellitus
Tianjin-GDM-PP: Tianjin Gestational Diabetes Mellitus Prevention Program
